# Supplementary material for: EUV-induced hydrogen desorption as a step towards large-scale silicon quantum device patterning
Source: Nat Commun. 2024 Jan 24;15:694. doi: 10.1038/s41467-024-44790-6 (PMC10808421; doi:10.1038/s41467-024-44790-6)
Supplement: Supplementary file 1 — Supplementary Information File [file 41467_2024_44790_MOESM1_ESM.pdf]

Supplementary Information: EUV-Induced Hydrogen Desorption As A Step Towards Large-Scale Silicon Quantum Device Patterning

Procopios Constantinou[*1,2,3], Taylor J. Z. Stock[1,4], Li-Ting Tseng[3], Dimitrios Kazazis[3], Matthias Muntwiler[3], Carlos A. F. Vaz[3], Yasin Ekinci[3], Gabriel Aeppli[3,5,6,7], Neil J. Curson[1,4], Steven R. Schofield[†1,2]

[1] *London Centre for Nanotechnology, University College London, WC1H 0AH, London, UK*

[2] *Department of Physics and Astronomy, University College London, WC1E 6BT, London, UK*

[3] *Paul Scherrer Institute, 5232 Villigen, Switzerland*

[4] *Department of Electronic and Electrical Engineering, University College London, London WC1E 7JE, UK*

[5] *Institute of Physics, Ecole Polytechnique Fédérale de Lausanne (EPFL), 1015 Lausanne*

[6] *Department of Physics, ETH Zürich, 8093 Zürich*

[7] *Quantum Center, Eidgenössische Technische Hochschule Zurich (ETHZ), 8093 Zurich, Switzerland*

[*] *procopios.constantinou@psi.ch*

[†] *s.schofield@ucl.ac.uk*

(Dated: Monday, 18 December 2023)

Supplementary Information Contents

Supplementary Note 1: STM dangling bond density determination 2

Supplementary Note 2: XPS fits of Si(001):H after non-monochromatic irradiation 3

Supplementary Note 3: Summary and model of EUV-induced hydrogen desorption.............. 4

Supplementary Note 4: Spectral profile of PEARL non-monochromatic irradiation............... 5

Supplementary References.. 5

Supplementary Note 1: STM dangling bond density determination

The dangling bond density is determined using the Gwyddion software package [1] by using two different threshold selection methods to find a range of dangling bond densities: (i) Otsu's threshold selection method [2] in Supplementary Figure 1a-b and (ii) basic threshold selection in Supplementary Figure 1c. Supplementary Figure 1 shows the results of three different STM images over the region corresponding to the maximum observed desorption after a 100 min non-monochromatic irradiation (Figure 1d in the main text). These methods determine the dangling bond density by finding the total relative area of the surface enclosed by the protrusions of dangling bonds on the surface, which can be treated as individual 'grains' in the STM images. It is important to state that the dangling bonds are not directly counted here, rather, the relative area encompassed by the protrusions of the dangling bonds in the STM images, so this offers at best an upper-bound estimate on the dangling bond density. A grain analysis was performed on the full STM image and a zoom-in around a region that contains no step edge. We find the range of measured dangling bond densities lies between 45% to 62%. Thus, to be conservative, the best estimate of the dangling bond density lies within (0.55 ± 0.10) ML.

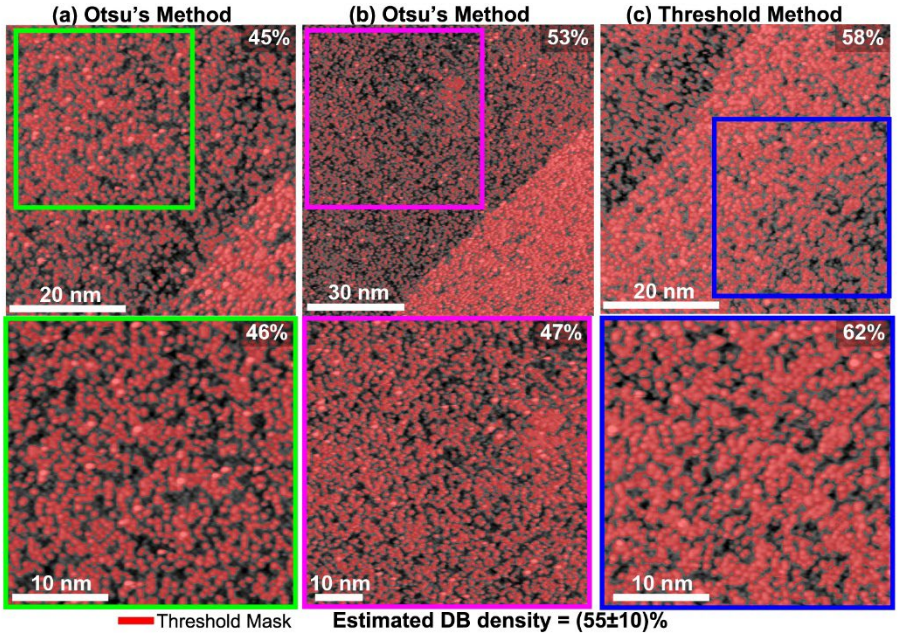

Supplementary Figure 1: STM grain analysis for dangling bond density determination. (a-b) Otsu's and (c) basic threshold method being employed to determine the relative dangling bond density. Three different STM images over the same region of the sample are analysed, along with a zoom-in over areas without a step-edge. The range of dangling bond densities lie within (0.55 ± 0.10) ML.

Supplementary Note 2: XPS fits of Si(001):H after non-monochromatic irradiation

Supplementary Figure 2 shows how the monohydride Si(001)/H photoelectron spectrum changes as a function of non-monochromatic irradiation time, where the fits are explicitly shown at five different times. This dataset is identical to that presented and discussed in the main text. Supplementary Figure 2a shows the results when no active heating/cooling was applied to the sample and, although not directly measured, we refer to these measurements as having a base temperature of 300 K (or room temperature). Supplementary Figure 2b shows measurements made when the sample was both irradiated and measured with XPS at a base temperature of 77 K. We see that in all instances, a very good fit to the data is obtained consistently across all irradiation times and temperatures. The changes in the spectra of Supplementary Figure 2 is consistent with the hydrogen terminated photoelectron spectrum transforming into the clean silicon spectrum, because of hydrogen desorption. One of the main differences in the fits shown in Supplementary Figure 2a,b is that the inhomogeneous broadening of the fit components is smaller at 77 K, relative to 300 K. At t = 0 min, the Gaussian FWHM of each component at 300 K is 295 meV, which reduces to 265 meV at 77 K. This reduction of 30 meV allows each chemically shifted component in the photoelectron spectrum to be more easily resolved at 77 K.

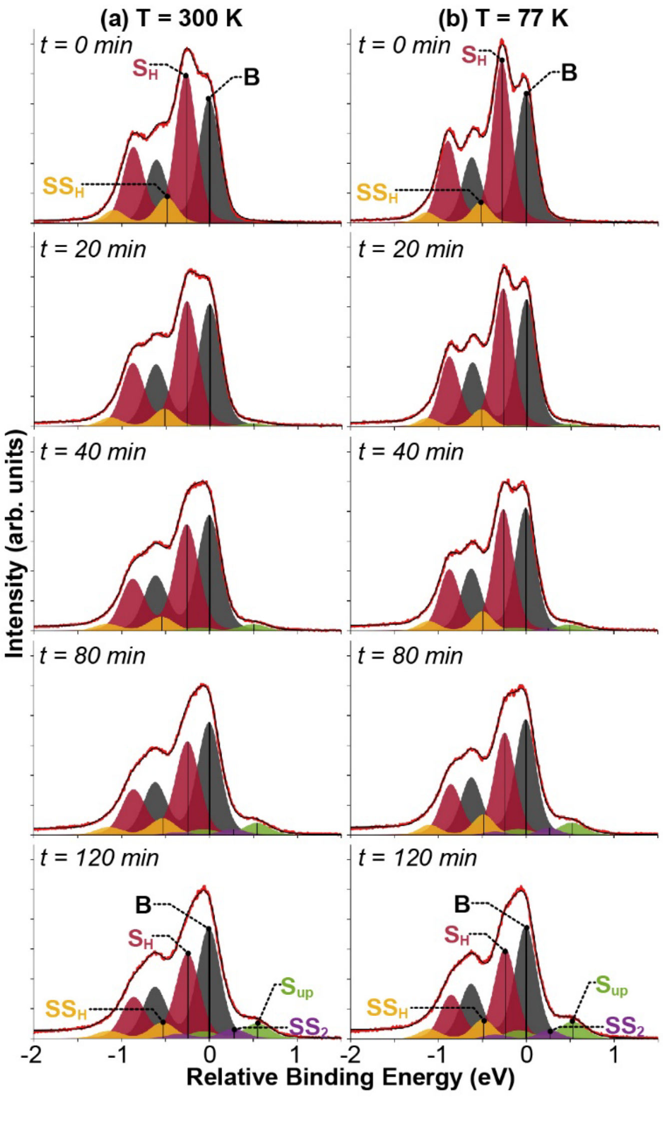

Supplementary Figure 2: Si 2*p* fits versus non-monochromatic irradiation time. (a-b) Series of fitted photoelectron spectra, equivalent to the data discussed in the main text, at a sample temperature of (a) 300 K (no active heating / cooling), (b) 77 K (with liquid nitrogen cooling). The total irradiation time is shown on the top left of each figure panel. The spectra are taken at $h\nu$ = 140 eV and θ = 60°. The five fitted components S_H, SS_H, S_{up}, SS_2 and B are all individually identified and color-coded, as discussed and shown in the main text. The solid red and black line show the raw and fitted data respectively, prior to background subtraction, whose maximum peak height is normalised to one. Each fitted component is plotted after being background subtracted.

Supplementary Note 3: Summary and model of EUV-induced hydrogen desorption

Supplementary Figure 3(a) shows a plot of the clean silicon density versus the photon irradiance. This plot is similar to Figure 2b in the main text, however, here it includes all data obtained from all photon irradiations at PEARL. We find that all data points lie in very good agreement with one another and within the confidence intervals of the fit. This establishes that the desorption mechanism is likely the same for all types of irradiations and that the principal determining factor is the photon irradiance.

The model solution for EUV-induced hydrogen desorption is shown in Supplementary Figure 3(b). The analytical solution (Equation 3 in the main text) and its derivative (Equation 4 in the main text) are shown as a solid black and blue line respectively. The model requires only one parameter to fit the data and shows that as hydrogen desorption progresses, the probability of further desorption decreases, slowing down the overall process. The clean silicon atom density gradually approaches 1 and, according to the model, to achieve a clean silicon atom density of 0.95 ML, a photon irradiance of 12×10^{20} ph/cm^2 is required; the largest we achieved during these experiments was 0.55 ML with a photon irradiance of 1.7×10^{20} ph/cm^2.

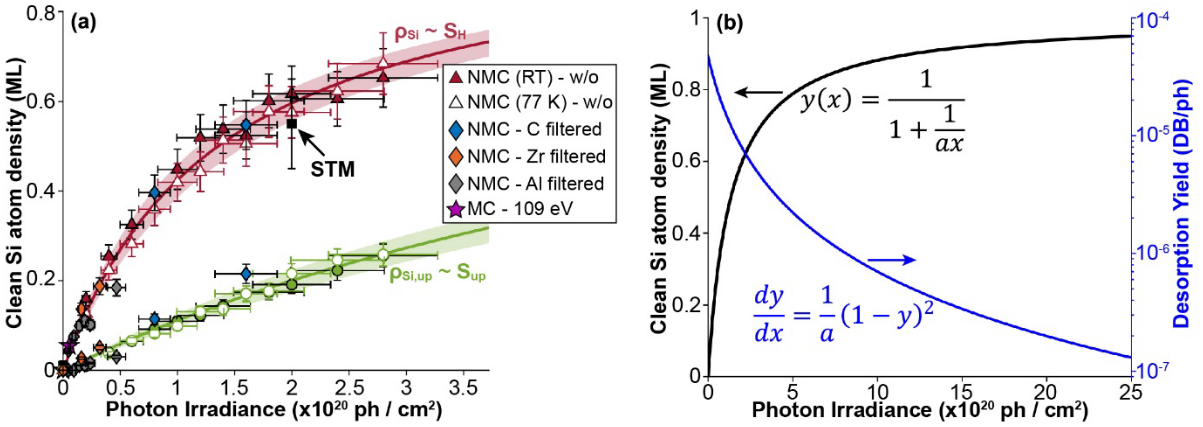

Supplementary Figure 3: Summary and model of hydrogen desorption measurements at PEARL. (a) Density of clean silicon atoms, ρ_{Si} (red) and up-buckled silicon atoms, $\rho_{\mathrm{Si,up}}$ (green) versus photon irradiance. Irradiations using non-monochromatic (NMC) light are shown with and without (w/o) the three different EUV filters, and monochromatic (MC) light at 109 eV. The labelled black data point shows the silicon atom density as measured from STM imaging. The symbols are from measured data and the lines show the best fit to the data. The NMC exposures are shown for both 77 K and RT, whereas the filtered NMC and MC exposures are at RT. The coloured areas around the fits represent the 90% confidence interval. A good agreement is found amongst all type of exposures. (b) Model solution for EUV-induced hydrogen desorption which is an exact solution to a first-order non-linear differential equation (Equation 4 in main text). The solution to the best fit of the data in (a) is shown on the left y-axis (in black) and its derivative is shown on the right y-axis (in blue). The right y-axis (in blue) is scaled to equal the desorption yield (or probability); as hydrogen desorption progresses, the probability of further desorption decreases, slowing down the overall process, with the clean silicon atom density slowly approaching 1.

Supplementary Note 4: Spectral profile of PEARL non-monochromatic irradiation

Non-monochromatic irradiation was performed at the PEARL beamline (bending magnet insertion device) by aligning the plane grating monochromator to its zero-order reflection, such that all photon energies passed through the beamline optics and were subsequently focussed onto the sample surface. Supplementary Figure 4 shows the calculated photon flux profile of the zero-order spectrum and its convolution with the transmittance of each x-ray filter. The spectral profile of the unfiltered non-monochromatic beam is a continuous distribution of photon energies, whose maximal photon flux is between 10 – 100 eV. Further, we see that by using the different x-ray filters, different transmission bands can be accessed: (i) Al has a maximum transmittance between 17 – 73 eV and above 350 eV; (ii) Zr has a maximum transmittance between 70 – 200 eV and above 700 eV; (iii) C has a maximum transmittance between 100 – 280 eV and above 350 eV. For comparison, the spectral profile of the PEARL monochromatic photon flux versus photon energy can be found in Ref. [3], which yields a brilliance that is two orders of magnitude smaller than the unfiltered non-monochromatic beam.

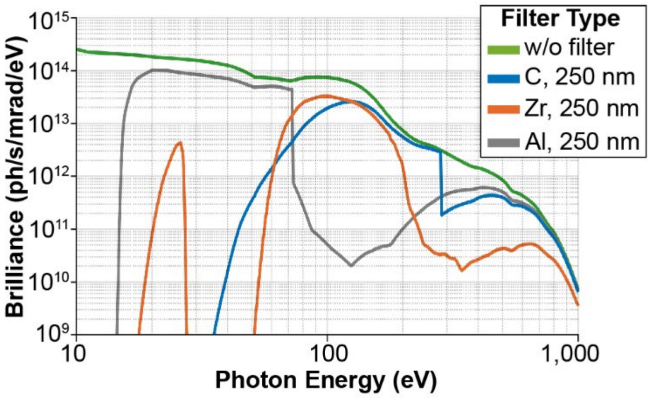

Supplementary Figure 4: Calculated non-monochromatic (zero-order) transmission spectrum of the x-ray filters. The calculated flux profile of the zero-order spectrum for non-monochromatic light at PEARL is shown, along with its convolution with each of the x-ray filters transmission profile.

Supplementary References

[1] D. Nečas and P. Klapetek, "Gwyddion: an open-source software for SPM data analysis," *Open Physics*, vol. 10, no. 1, pp. 181–188, Jan. 2012, doi: 10.2478/s11534-011-0096-2.

[2] N. Otsu, "A Threshold Selection Method from Gray-Level Histograms," *IEEE Trans Syst Man Cybern*, vol. 9, no. 1, pp. 62–66, Jan. 1979, doi: 10.1109/TSMC.1979.4310076.

[3] M. Muntwiler *et al.*, "Surface science at the PEARL beamline of the Swiss Light Source," *J Synchrotron Radiat*, vol. 24, no. 1, pp. 354–366, 2017, doi: 10.1107/S1600577516018646.
